# Research Progress on the Application of Natural Medicines in Biomaterial Coatings

**DOI:** 10.3390/ma17225607

**Published:** 2024-11-16

**Authors:** Yanchao Wang, Huimin Duan, Zhongna Zhang, Lan Chen, Jingan Li

**Affiliations:** School of Materials Science and Engineering, Zhengzhou University, Zhengzhou 450001, China; wangyancaho@gs.zzu.edu.cn (Y.W.); duanhuimin@gs.zzu.edu.cn (H.D.); zhongna_zhang@gs.zzu.edu.cn (Z.Z.)

**Keywords:** natural medicines, biomaterials, coatings, applications, orthopedic implants, cardiovascular and cerebrovascular stents, wound dressings

## Abstract

With the continuous progress of biomedical technology, biomaterial coatings play an important role in improving the performance of medical devices and promoting tissue repair and regeneration. The application of natural medicine to biological materials has become a hot topic due to its diverse biological activity, low toxicity, and wide range of sources. This article introduces the definition and classification of natural medicines, lists some common natural medicines, such as curcumin, allicin, chitosan, tea polyphenols, etc., and lists some biological activities of some common natural medicines, such as antibacterial, antioxidant, antitumor, and other properties. According to the different characteristics of natural medicines, physical adsorption, chemical grafting, layer-by-layer self-assembly, sol–gel and other methods are combined with biomaterials, which can be used for orthopedic implants, cardiovascular and cerebrovascular stents, wound dressings, drug delivery systems, etc., to exert their biological activity. For example, improving antibacterial properties, promoting tissue regeneration, and improving biocompatibility promote the development of medical health. Although the development of biomaterials has been greatly expanded, it still faces some major challenges, such as whether the combination between the coating and the substrate is firm, whether the drug load is released sustainably, whether the dynamic balance will be disrupted, and so on; a series of problems affects the application of natural drugs in biomaterial coatings. In view of these problems, this paper summarizes some suggestions by evaluating the literature, such as optimizing the binding method and release system; carrying out more clinical application research; carrying out multidisciplinary cooperation; broadening the application of natural medicine in biomaterial coatings; and developing safer, more effective and multi-functional natural medicine coatings through continuous research and innovation, so as to contribute to the development of the biomedical field.

## 1. Introduction

**Figure 1:**
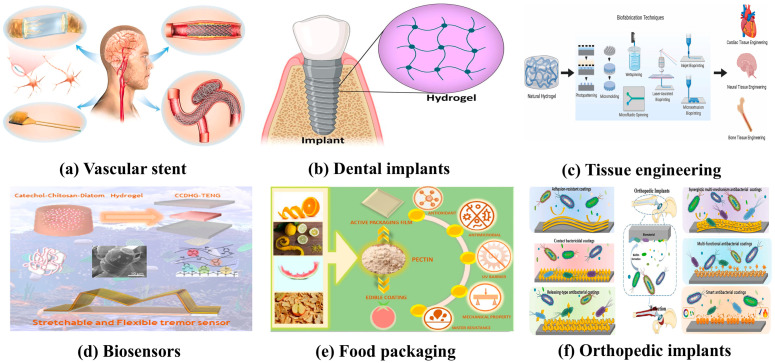
Some applications of biomaterial coatings: (**a**) Mg-based alloys have been used in neuroscience as filaments within nerve conduits to accelerate nerve regeneration, the nerve electrode, devices for neural recording and monitoring, and stents for carotid artery stenosis and aneurysm treatment [32]; (**b**) the incorporation of compounds such as titanium dioxide (TiO_2_), dopamine, fluorine-substituted hydroxyapatite (FHA), tetraethyl orthosilicate (TEOS), and silica nanoparticles (SNs) into the hydrogel structure can improve the biocompatibility, stability, and peripheral inflammation of implants [33]; (**c**) the prepared hydrogels are used for cardiac, nervous, and bone tissue engineering [34]; (**d**) catechol chitosan diatom hydrogel (CCDHG) was developed for use in TENG electrodes, and m-type defibrillation sensors were developed based on CCDHG-TENG to evaluate low-frequency motion in patients with Parkinson’s disease [35]; (**e**) plant-based multi-confectionery gums can be used to produce polymer films for active packaging [36]; (**f**) an antimicrobial coating can be built on the surface of orthopedic implants [37].

Biomaterial coatings are increasingly used in medical and biological tissue engineering, ranging from diagnosis and treatment to tissue repair and organ replacement. In the field of diagnostics, sensors and detection reagents made of biomaterials can quickly and accurately detect biomarkers, providing strong support for the early diagnosis of diseases [1,2,3]. In terms of treatment, the application of biomaterials such as drug carriers and interventional devices has significantly improved the therapeutic effect and reduced side effects [4,5]. In tissue engineering and regenerative medicine, biomaterials play a key role [6,7]. For example, scaffold materials used for bone repair can guide the growth of bone cells and promote bone tissue regeneration [8,9,10]. Biomaterials such as artificial blood vessels and heart valves in the cardiovascular field help to restore normal blood circulation [11,12,13,14]. In addition, biomaterial coatings have also been used in ophthalmology, stomatology, neurosurgery, and other fields, making great contributions to improving the quality of life of patients [15,16,17,18,19]. Figure 1 illustrates some of the applications. Biomaterial coatings are an effective means to significantly improve the performance and biocompatibility of biomaterials. However, there are still some problems with traditional biomaterial coatings. The risk of implant infection remains, due to limited antimicrobial performance and the difficulty of effectively countering the growing problem of bacterial resistance; the anticoagulant effect is not ideal, and thrombotic complications may still occur during long-term use. In addition, the biocompatibility of some coatings remains to be improved, which may cause a local inflammatory response or immune rejection. In addition, traditional coatings may use toxic chemical agents in the preparation process, posing a potential threat to the environment and human health. In the complex physiological environment of living organisms, the coating may be subject to mechanical stress, chemical corrosion, biodegradation, and other factors, which will lead to the peeling or degradation of the coating, which will affect the performance and service life of the coating [20,21,22]. For biomaterial coatings with drug release, how to accurately control the release rate and the amount of release is a problem, and a release that is too slow may not achieve therapeutic effect. Releasing it too quickly may lead to increased side effects of the drug [23,24,25].

In view of the above-mentioned problems in traditional biomaterial coatings, natural medicines are gradually gaining attention as a potential solution. Natural medicine refers to drugs of natural origin, unmodified or synthesized and used under the guidance of modern medical theories, including pharmaceutical ingredients from plants, animals, and microorganisms. Natural medicines are rich in bioactive ingredients and diverse mechanisms of action, such as antibacterial, anti-inflammatory, antioxidant, etc. The application of natural medicines to biomaterial coatings is expected to overcome the limitations of traditional coatings. For example, some natural medicines have potent antimicrobial activity and are able to effectively inhibit the growth of multiple drug-resistant bacteria, providing new strategies for preventing implant infections [26]. At the same time, the anti-inflammatory and antioxidant properties of natural medicines can regulate the body’s immune response, reduce inflammatory damage, and improve biocompatibility [27,28,29,30]. In addition, natural medicines usually have low toxicity and good biodegradability [31], which is in line with the development concept of green environmental protection. The role of natural medicine is equivalent to building a barrier on the surface of the scaffold to realize the functionalization of the stent. At present, the application of natural medicine in the coating of biological materials has made some progress. Researchers have explored the application of a variety of natural medicines in different types of biomaterial coatings. In terms of antimicrobial coatings, natural medicines such as berberine, curcumin, and allicin have been used to prepare coatings with antimicrobial functions and have shown good results in in vitro experiments and animal models. These coatings can effectively inhibit the attachment and growth of bacteria, reducing the incidence of infection. In terms of anticoagulant coatings, the application research of natural drugs such as salvia extract and hirudin is also constantly advancing, providing a new way to improve the anticoagulant performance of cardiovascular devices. In the coating that promotes tissue regeneration, natural medicines such as collagen and chitosan are used to promote cell adhesion, proliferation and differentiation, and accelerate the process of tissue repair and regeneration. There is also the use of polymer coatings to encapsulate natural medicines to achieve controlled release of drugs under certain conditions.

While the application of natural medicines in the coating of biomaterials is still in the research stage, there are some issues that need to be addressed. For example, the stability and controlled release of natural medicines need to be further optimized, and the preparation process of coatings needs to be perfected to achieve large-scale production and clinical application. However, with the continuous deepening of research, it is believed that natural medicine will have broad application prospects in the field of biomaterial coating, and more new natural medicines will be discovered, and multidisciplinary cross-integration will promote the progress of technology. Also, the specific appropriate drug coating is selected for treatment according to the patient, so as to make a greater contribution to the improvement of medical outcomes and the quality of life.

## 2. Preparation Method of Coating

Different natural medicines possess unique properties, functional groups, and mechanisms, while biomaterials exhibit distinct characteristics. How does a natural medicine bind to the surface or within another material? Several common methods are identified through a review of the literature, including physical adsorption, chemical grafting, layer-by-layer self-assembly, the sol–gel method, and electrospinning.

### 2.1. Physical Adsorption

Principle: The use of intermolecular van der Waals force, electrostatic action, etc., to adsorb natural drugs to the surface of biomaterials. The biomaterial is soaked in a solution containing natural medicines, and after a certain period of incubation, the drugs are adsorbed on the surface of the material. For example, the titanium alloy material is soaked in the solution of tea polyphenols, and the tea polyphenols form an antioxidant coating on the surface of the titanium alloy through physical adsorption [38]. Clindamycin was loaded onto cerium dioxide nanoparticles (CNPs) by the physical adsorption method [39]. Polyacrylamide (PAM) and graphene oxide (GO) were combined by the physical adsorption method, and then loaded with clove essential oil to make composite nanomaterials for antibacterial packaging [40]. Liu Sisi et al. [41] first prepared chitosan/silk fibroin nanofiber multilayer membranes through self-assembly layer by layer, and loaded the antibacterial drug berberine into the multilayer membrane by the physical adsorption method, thereby preparing berberine–chitosan/silk-fibroin nanofiber multilayer membranes. Lubna Shahzadi et al. [42] prepared chitosan hydrogel by the freezing gel method, and then soaked it in a solution of thyroxine completely adsorbed into the hydrogel, and then dried it to form a hydrogel. Although the physical adsorption method is relatively simple and low-cost, the binding force between the coatings is weak, and the loading of drugs on the coating is limited.

### 2.2. Chemical Grafting

Natural medicines are covalently bound to active groups on the surfaces of biomaterials through chemical reactions. Covalent bonds are employed to secure the surface of the material, which is first activated to introduce active groups. These groups subsequently react with natural drugs that have undergone specific chemical modifications. However, the reaction conditions can be harsh, and the construction process is often time-consuming and costly, complicating actual production [43]. Additionally, the involvement of certain groups within the drug molecule in the formation of covalent bonds may alter the overall structure of the drug, potentially impacting both the biological activity of the material and the drug itself [44]. Examples of chemical grafting are provided in the literature, as detailed in Table 1.

### 2.3. Layer-by-Layer Self-Assembly

The layer-by-layer self-assembly technology is based on the interaction force between different molecules, so that the molecules are alternately deposited on the base to form a multi-layer structure. These interaction forces include electrostatic interactions, hydrogen bonds, coordination bonds, van der Waals forces, etc. It is simple, easy to operate, and the thickness can be controlled, with good adaptability. Tan et al. [50] used the electrostatic interaction between sodium alginate (SA) and chitosan (CS) to form a stable modification layer on the surface of liposomes. Zhang et al. [51] used polyurethane as the base material to alternately deposit heparin and chitosan onto a decellularized scaffold (PU/DCS).

### 2.4. Sol–Gel Method

Principle: The hydrolysis and polycondensation reaction of the precursor in solution are used to form a gel, in which the natural medicine is embedded, and then applied to the surface of the biomaterial. Natural medicines are mixed with sols and subjected to steps such as gelation, drying, and heat treatment to form a coating. Violav et al. [52] used tetraethyl orthosilicate as a precursor of silica by the sol–gel method, in which polyvinyl alcohol was dissolved in ethanol and added to the silicon matrix, and green oxalic acid was slowly added to the mixed solution and, finally, an organic–inorganic material was prepared. The sol–gel method can be used to prepare nanoparticles and encapsulate drugs to achieve targeted drug delivery [53]. The porosity of the coating prepared by this method is controllable, and the coating uniformity is good, but the reaction conditions need to be strictly controlled.

### 2.5. Electrospinning

In the electrospinning process, a solution or melt containing natural medicines and polymers is placed in a syringe with a metal needle attached to the front end of the syringe to apply a high voltage between the needle and the receiving device. Under the force of an electric field, a polymer solution or melt forms a Taylor cone from the tip of the needle and ejects a charged trickle. As these streams fly towards the receiving device, the solvent volatilizes or the melt cools and solidifies, forming continuous fibers that are deposited on the receiving device, resulting in a fiber coating containing natural medicines [54,55]. For example, curcumin is mixed with a polylactic acid–glycolic acid copolymer (PLGA) to prepare an electrospinning solution [56]. The electrospinning process was used to obtain a coating of PLGA fibers containing curcumin. Curcumin has biological activities, such as anti-inflammatory and antioxidant activities, and this coating can be applied to tissue-engineered scaffolds to promote tissue repair and regeneration.

## 3. Natural Medicines and Biomaterials Form Coatings and Their Applications

Loading coatings on the surface of biomaterials not only changes the physical and chemical properties of the materials but also affects their biocompatibility and biointegration capabilities. The implantation of biomaterials with surface coatings can improve the compatibility of materials, reduce immune responses, better bind to cells and tissues, and reduce postoperative complications and the need for reoperation. It can also enhance the mechanical properties and corrosion resistance of the material. In addition, surface coatings can endow biomaterials with specific functions, such as using natural medicines with antibacterial, anti-inflammatory, and tissue-regeneration properties to achieve functional therapeutic effects when combined with biomaterials. Here, some common natural medicines that work in combination with biomaterials based on their biological properties are used, such as by forming an antimicrobial coating that inhibits the growth of bacteria and can reduce the risk of infection. Combining these with biomaterials facilitates the reduction of inflammatory responses based on the resistance properties of natural medicines. According to the needs of functionalization, the controlled release of drugs can be realized to achieve effective treatment.

### 3.1. Curcumin

Curcumin is a natural polyphenolic compound, often found in turmeric rhizomes, the chemical formula of curcumin is C_21_H_20_O_6_. Curcumin can not only be used as a spice in food, but also in medicine and health, because of its multiple biological activities. Curcumin has antioxidant, anti-inflammatory, and antibacterial properties, and has a certain protective effect on Alzheimer’s disease and Parkinson’s disease [57,58,59,60,61].

Studies have proved that curcumin has broad-spectrum antibacterial activity, and has an antibacterial effect on both Gram-positive and -negative bacteria [62,63]. Curcumin can destroy the structure and permeability of bacterial cell walls and cell membranes, interfere with the synthesis of bacterial nucleic acids, proteins and fatty acids, and inhibit the formation of bacterial biofilms [62,63,64,65]. Kandaswamy et al. [66] loaded curcumin and berberine chloride into polymer nanofibers, and curcumin and berberine chloride were able to reduce the production of extracellular polymeric plasma (EPS), which would damage the protective barrier of the biofilm and lead to the instability of the overall structure of the biofilm. Nanofibers have a significant antibacterial effect on methicillin-resistant Staphylococcus aureus (MRSA). W et al. [67] encapsulated montmorillonite (MMT), L-malic acid (LMA) and curcumin (Cur) into a bacterial cellulose (BC) matrix to prepare multifunctional nanofilms; curcumin can interact with bacterial proteins and bind to deoxyribonucleic acid molecules to destroy the cell wall and cell membrane of bacteria [64], thereby destroying the cell membrane and inhibiting the growth of Staphylococcus aureus and *Escherichia coli*. Simin et al. [68] prepared hydroxyapatite–gelatin–curcumin nanofiber composites by electrospinning, which were released from the composites through a two-step mechanism, which would enter the bacteria and cause damage to the bacteria by the interaction between bacterial proteins and DNA. Li [69] uses a single-step precipitation process to load curcumin onto the surface of hydroxyapatite (HA), and curcumin-functionalized HA can inhibit the cell attachment of Staphylococcus aureus and pseudomonas aeruginosa, which can be used as an antimicrobial agent to control the risk of postoperative infection. Curcumin is loaded on a polyvinyl alcohol/cellulose nanocrystal (PVA/CNC) membrane, which is concentration-dependent on curcumin, with decreased activity of breast cancer cells and liver cancer cells at 8 mg/mL. A chitosan/curcumin multilayer coating was loaded on PEEK/BG/h-BN by electrophoretic deposition method, and the antimicrobial activity of the coating was tested according to the optical density, and it was found that the multilayer coating had a good antibacterial effect on Gram-positive and Gram-negative bacteria, because curcumin changed the permeability of the bacterial cell membrane and entered the bacteria to destroy DNA activity, resulting in the death of bacteria [70], so the chitosan/curcumin-PEEK/BG/H-BN coating can be used for antiseptic orthopedic implants [71].

**Figure 2:**
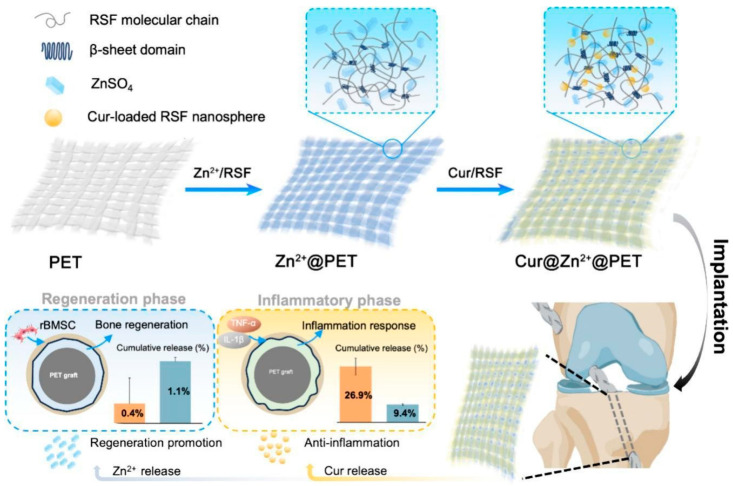
A dual-loaded multi-layered RSF coating with curcumin and Zn^2+^ on PET grafts, which followed a time-programmed pattern of drugs release, could intervene anti-inflammatory and tissue regeneration in a time-matched way, and ultimately improve graft–host integration [77].

Curcumin’s ability to inhibit the production of inflammatory cytokines, such as interleukin-1 (IL-1) and interleukin-1 (IL-6) and TNF-α, as well as the significant inhibition of other inflammatory mediators such as nuclear factor (NF-κB), cyclooxygenase 2 (COX2), and matrix metalloproteinase-9 (MMP 9), can reduce inflammation at the implant site. Li et al. [72] prepared polychin–curcumin-loaded PCL-PEG nanofibers by electrospinning technology, and found that the drug-loaded nanofiber materials affected the expression of IL-6, MMP-2, TIMP-1, TIMP-2 and iNOS genes in multiple stages of wound healing, and could shorten the time of wound healing. Curcumin-loaded lipid–poly(lactic-*co*-glycolic acid) (PLGA; Cur@MPs) hybrid microparticles (MPs) fabricated using an oil-in-water single emulsion method are embedded into gelatin-based scaffolds. It was found that the scaffold can regulate the Nrf2/HO-1 signaling axis, inhibit the secretion of pro-inflammatory factors by macrophages, and inhibit the migration and angiogenesis of vascular endothelial cells, which can be used to prevent complications after corneal transplantation. Li et al. [73], found that the novel material can activate the Nrf2/ARE pathway, inhibit inflammation, reduce reactive oxygen species to reduce cellular oxidative stress, and can be used in wound dressings to promote wound healing. Xian mou Fan et al. [74] prepared a new type of polyvinyl alcohol (PVA)-chitosan (CS)/sodium alginate (SA)-Cur hydrogel (PCSA), which can convert pro-inflammatory M1 macrophages into anti-inflammatory M2 macrophages, and was found to be able to clear ROS, down-regulate IL-1β, and up-regulate CD31 expression in a full-thickness wound model of rat diabetes, which can accelerate wound healing and promote angiogenesis and collagen deposition. The hydrogel can be used for diabetic wound healing. Nanoparticle curcumin was loaded onto the surface of titanium implants for modification, and curcumin was found to enhance the nuclear translocation and DNA binding activities of Runx2 and osterix, which can be used to enhance the expression of osteoblast and bone-matrix protein genes [75]. First, hydroxyapatite was used to modify the orthopedic implants of titanium and alloys to enhance the biological activity, and then curcumin (Cur) and epigallocatechin gallate (EGCG) were added, and it was found that the release of curcumin and EGCG could promote the growth of osteoblasts, and curcumin could enhance the expression of osteocalcin and Runx2 to promote the proliferation and differentiation of osteoblasts, which can be used to treat bone tumors [76]. First, Gao et al. [77] designed a double-layered drug-loaded RSF coating on the PET surface and encapsulated the anti-inflammatory drug (curcumin) and tissue release factor (Zn+) in the REF coating, as shown in Figure 2. The release of curcumin and Zn+ can inhibit the inflammatory response and promote tissue regeneration.

Zhang Ling et al. [78] synthesized monocarbonyl curcumin analog A2 and found that curcumin analog A2 can increase the level of ROS in endothelial cells, thereby inducing cell death and exerting antiangiogenic activity. The curcumin analog A13 activates the Nrf2/ARE pathway, thereby reducing oxidative damage to ameliorate myocardial fibrosis in diabetic rats [79]. Chen et al. [80] evaluated the anti-tumor effect of WZ26 in both CCA cells and a CCA xenograft mouse model. WZ26 significantly increased ROS in the CCA cancer cells, thereby inducing mitochondrial apoptosis and inhibiting cancer cell growth both in vitro and in vivo.

### 3.2. Allicin

Allicin is a sulfur-containing organic matter extracted from garlic, with a strong garlic odor; its chemical name is diallyl thiosulfinate, its chemical formula is C_6_H_10_S_20_, and its functional groups include the sulfide bond (-S-) and sulfonate group (-SO-). Allicin inhibits and kills a variety of bacteria, fungi, and viruses, as well as reducing the risk of atherosclerosis by regulating blood lipids to reduce blood cholesterol and triglyceride levels. It inhibits the aggregation of platelets, reduces blood viscosity, and prevents the formation of thrombosis; it can scavenge free radicals in the body, reduce oxidative stress to cells, and delay aging [81,82,83,84,85,86].

Allicin has a broad-spectrum antibacterial effect on a variety of bacteria, such as Gram-positive and -negative bacteria, and the application of allicin to wound dressings can stimulate the proliferation of fibroblasts and the synthesis of collagen, and promote the growth of granulation tissue. Pang et al. [87] prepared a multifunctional nanoplatform for Allicin@ZIF-8/AgNPs, and the release of allicin can reduce the level of ROS to inhibit the production of cytokines and promote the production of collagen fibers, stimulate the production of new blood vessels, and improve the speed of wound healing. Wang et al. [88] prepared a micellar composite hydrogel loaded with allicin; the release of allicin can be antibacterial, reduce ROS, and promote cell metabolism. According to the constructed rat full-thickness skin injury model, it was found that the wound healing time was shortened, and the color of granulation tissue was lightened by HE staining. Therefore, allicin-loaded micellar composite hydrogel can be used as a wound dressing to accelerate wound healing. Liu Yongxu et al. [89] added allicin (All) to chitosan (CS)/polyvinyl alcohol (PVA)/graphene oxide (GO) composites, and used electrospinning technology to prepare nanofiber membranes, which have strong antibacterial effects according to antibacterial experiments and allicin in vitro release surface nanofiber membranes, which can be used for antibacterial wound dressings and tissue engineering.

**Figure 3:**
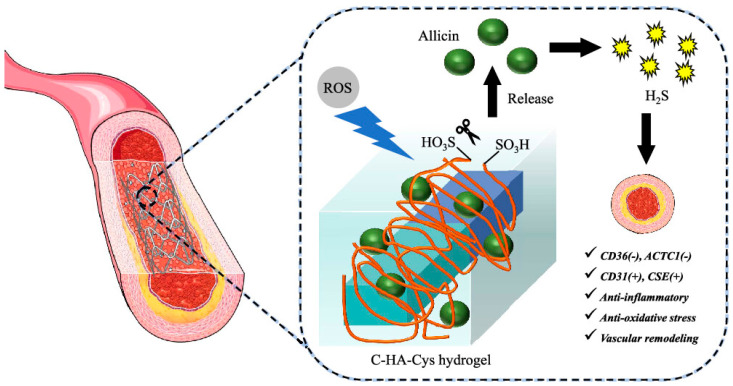
C-HA-Cys hydrogel coatings were prepared by an amide reaction using catechol hyaluronic acid (C-HA) and cystine (Cys). The H_2_S-releasing donor allicin is loaded into the hydrogel to form a smart biomimetic coating [95].

Allicin can upregulate alkaline phosphatase (ALP) and osteocalcin (BGLAP) to promote the proliferation and differentiation of mesenchymal stem cells (hMSCs), and the release of allicin was found to promote collagen formation in a rat distal femur model [90]. Allicin was applied to the treatment of mouse models of cerebral hemorrhage, and it was found that the injection of allicin had a neuroprotective effect, which could inhibit the accumulation of cells around the hematoma and reduce axonal damage [91]. It also inhibits pro-inflammatory factors in the brain, such as interleukin 6 and C-X-C motif ligand 2 in the brain. Therefore, it can be applied to the treatment of intracerebral hemorrhage disorders. The nanofiber wound dressing was prepared by using polycaprolactone (PLC) and silk fibroin, and the antibacterial effect could be used to treat burns because the water contact angle was reduced to 85.5° and the Young’s modulus was increased to 5.12 MPa after the addition of allicin [92]. A hydroxyapatite drug delivery system loaded with allicin was prepared, and polycaprolactone (PCL) increased the kinetics of allicin release, increasing the release of allicin from 35% to 70%, and antibacterial experiments found that allicin had an excellent antibacterial effect on Staphylococcus aureus, and had good compatibility with osteoblasts, and could be used as a bone-tissue engineering scaffold to exert antibacterial, drug release, and compatibility characteristics [92]. In the treatment of a rat diabetic wound model, it was found that the loading of allicin dressing improved the wound closure rate, and the epithelial cell thickness and collagen deposition were higher than those of the non-allicin dressing group, so the clinical application of a allicin/chitosan/polyvinyl alcohol dressing in diabetic wounds has great potential [93]. Using a 3D-printed bone-tissue engineering scaffold to load curcumin and allicin, the drug release can promote the proliferation of osteoblasts, reduce the activity of osteoclasts, and enhance osseointegration, which can be used for low-load bone-tissue engineering and dental applications. Using the porous structure of the white flesh of grapefruit peel as a scaffold, a new type of natural antibacterial patch was prepared by using the antibacterial properties of allicin, and it was found that the natural antibacterial patch could effectively kill Gram-negative and -positive bacteria, and also had anti-skin infection activity [94]. Han et al. [95] prepared C-HA-Cys hydrogel coatings by an amide reaction using catechol hyaluronic acid (C-HA) and cystine (Cys). The H2S-releasing donor allicin is loaded into the hydrogel to form a smart biomimetic coating (this is shown in Figure 3). Loading the surface of the vascular interventional device with a C-HA-Cys hydrogel coating enables drug release and alters the lesion microenvironment, thereby preventing stent restenosis.

### 3.3. Berberine

Berberine is an alkaloid extracted from various plants such as Coptis chinensis and Phellodendron chinensis. The molecular formula is C_20_H_18_NO_4_, containing quaternary ammonium salt functional groups, and the taste is extremely bitter. Berberine can exert neuroprotective effects by upregulating the survival of PI3K/Akt/Bcl-2 cells and the Nrf2/HO-1 antioxidant signaling pathway [96]. Berberine can lower blood glucose, maintain homeostasis, inhibit the release of inflammatory mediators (TNF-α, NF-κB, phosphorylated NF-κB-p65, cox-2 and iNOS) [97], improve atherosclerosis, protect cardiovascular and cerebrovascular vessels [98], and interfere with bacterial metabolic processes, such as destroying the structural integrity of bacteria to inhibit bacterial proliferation.

Berberine has a wide range of pharmacological effects [99,100,101], such as adding berberine to the surface coating of medical devices (urinary catheters, orthopedic implants), which can effectively inhibit the reproduction of bacteria on the surface of materials. The application of berberine in the coating of tissue-engineered materials can accelerate wound healing and tissue integration; medical devices that come into contact with blood (such as cardiac and cerebrovascular stents) inhibit the aggregation of platelets and the activity of clotting factors, thereby reducing the risk of thrombosis; it can also be used in combination with other bioactive ingredients to prepare multifunctional composite coatings. Zhong Xiaojun et al. [102] isolated Coptisine, an alkaloid from Coptis chinensis, used the fluorescent probe PI to detect it, and found that the PI signal in Cryptococcus cells treated with Coptisine increased, and the surface Coptisine changed the permeability of the bacterial membrane, resulting in the leakage of key substances in the cell and cell death. In addition, Coptisine can also lead to an excessive accumulation of ROS in Cryptococcus cells, and lipid peroxidation targets mitochondria and nuclei, among others, and ultimately leads to cell death. Dong et al. [103] found that berberine could increase the expression of Runx2, activate the typical Wnt/β-catenin signaling pathway, and promote the osteogenic differentiation of MSCs. At the same time, berberine can alleviate hydrogen peroxide (H_2_O_2_)-induced apoptosis of rat bone marrow mesenchymal stem cells in vitro. Berberine is combined with nano-hydroxyapatite/chitosan bone cement to prepare an antibiotic drug delivery system for bone healing [104]. E et al. [105] loaded berberine into a PCL/Gel scaffold and evaluated the healing process after stent implantation in a rat skull defect model, and found that the hydrophilicity of berberine was improved, which could promote the proliferation of fibroblasts and osteoblasts, and provide the possibility for bone-healing applications. A hydrogel was developed by combining the hypolipidemic properties of silk fibroin and the antioxidant properties of melanin with the therapeutic effect of berberine, which can be used for the wound healing of diabetic wounds [106]. The release of berberine in the hydrogel is able to protect the wound from bacterial infection. The hydrophobic and cationic nature of berberine makes microbial cell membranes permeable and stably intercalated with DNA. It inhibits the synthesis of DNA and the protein of bacteria, and has a good antibacterial effect on bacteria.

**Figure 4:**
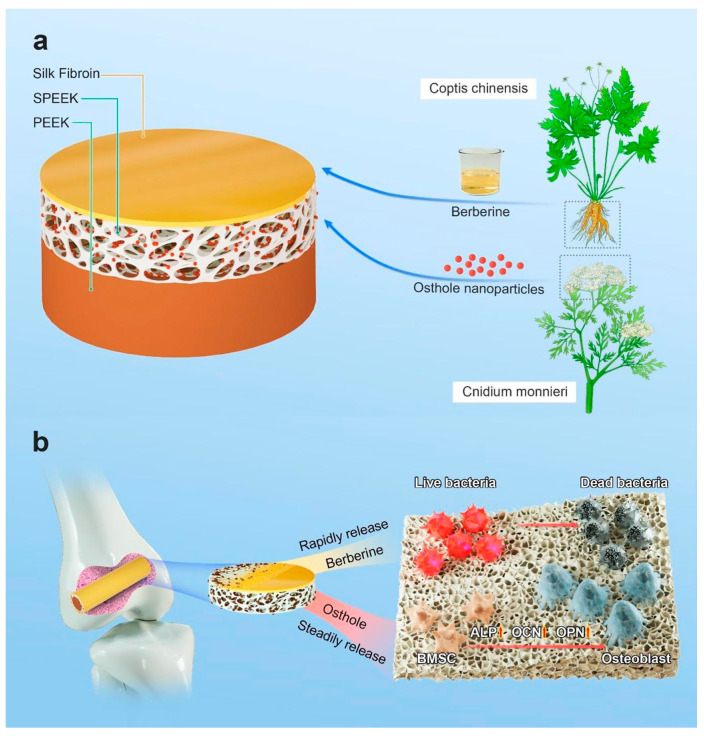
Polyetheretherketone (PEEK), which can be used for orthopedic implants, is selected to form a spongy three-dimensional structure on the surface through a sulfonation reaction and embedded osthole nanoparticles with osteogenic activity. The silk fibroin–berberine coating with antimicrobial function is loaded on the surface of the material [109]. (**a**) Composition of the coating; (**b**) The antibacterial and osteogenic functions of the coating.

Cellulose acetate–hyaluronic acid electrospun fibers loaded with berberine were manufactured by electrospinning [107], which can reduce the production and release of inflammatory factors, block the activation of inflammatory signaling pathways such as NF-kB, and can be used as a wound dressing to improve wound-healing time [108]. By sulfonation on the surface of polyetheretherketone (PEEK) of orthopedic implants to form a spongy three-dimensional structure, osteogenic active Cnidium monnieri nanoparticles were added, and a silk fibroin–berberine coating was applied to the surface to increase antibacterial function. In rat femur implantation experiments, it was found that Ost Ber @SPEEK promoted the activity of osteoblasts and prevented bacterial infection. Functionalized PEEK-based implants were prepared for use in orthopedics to promote osteogenic differentiation (Figure 4) [109]. Titanium and titanium alloys have good corrosion resistance and biocompatibility applied to orthopedic implants, but because titanium and titanium alloys do not have antibacterial and osteogenic properties, the graphene oxide (GO) of berberine (Ber) will be loaded on the titanium surface; that is, Ber-GO@Ti, Ber-GO@Ti has strong antibacterial activity against Staphylococcus aureus [104], which can promote the osteogenic differentiation of MC3T3-E1 cells, and there are no toxic and inflammatory cells in vivo experiments. This versatile coating can be applied to orthopedic implants to function. Two-dimensional Ti_3_C_2_ and berberine were loaded into the 3D-printed biphasic calcium phosphate (BCP) scaffold [110], both Ti_3_C_2_ and BBR had good antibacterial properties, Ti_3_C_2_ was beneficial to the generation of new bone, and the functionalized BCP scaffold was used to treat the problems of bone-defect infection according to the antibacterial and osteogenic properties.

### 3.4. Tea Polyphenols

Tea polyphenols are a general term for polyphenols in tea, the main components of which are catechins, which belong to the flavanols and have a basic structure of 2-phenybenzopyran. Common catechins include epicatechin (EC), epigallocatechin (EGC), epicatechin gallate (ECG), and epigallocatechin gallate (EGCG), among others, as shown in the figure. The hydrogen atom, with multiple phenolic hydroxyl groups, is easy to react with free radicals and terminate the chain reaction of free radicals, as it has strong antioxidant properties. Tea polyphenols can inhibit inflammatory factors (such as tumor necrosis factor-α (TNF-α) and interleukin-6 (IL-6), etc., and reduce the inflammatory response after stent implantation. It can regulate the function of vascular endothelial cells, increase the release of nitric oxide, inhibit the aggregation of platelets and the formation of thrombosis, and reduce the risk of cardiovascular and cerebrovascular diseases [111,112].

Zhang et al. [113] evaluated the leakage of protein, DNA, and K^+^, and found that tea polyphenols (TPs) destroyed the cell membrane, resulting in the loss of intracellular components, and finally led to cell death. Using the good antimicrobial activity of tea polyphenols, it is encapsulated into electrospun fibers and can be used for wound healing. As a biodegradable metal material, magnesium alloy is susceptible to corrosion and premature leakage of implants, Zhang Bo et al. [114] loaded the active factor epigallocatechin gallate (EGCG) in green tea onto the magnesium alloy (AZ31) matrix through layer by layer self-assembly method, and the coating provided an anti-corrosion barrier for bare AZ31. After a series of experiments, it was found that the surface of the EGCG/Mg coating has good corrosion resistance and superhydrophilicity, which can inhibit the denaturation of fibrinogen, improve hemocompatibility, prolong coagulation time and reduce thrombosis, and can be applied to cardiovascular implants. Zhang et al. [114] constructed an anodizing/tea polyphenol composite layer on the surface of AZ31 magnesium alloy, and the EGCG selected not only improved the corrosion resistance of the magnesium matrix, but also improved the surface modification of the medical magnesium alloy on the implant according to its antioxidant and anti-inflammatory properties.

Ren et al. [115] co-mixed tea polyphenols with high molecular weight polyethylene for joint replacement prostheses, which use tea polyphenols to produce excess reactive oxygen species (ROS) to destroy the bacterial membrane structure and reduce bacteria-induced inflammation in the body. Li Huimin et al. [116] immersed the porous structure of carboxymethyl chitosan/sodium alginate/tea polyphenol into the impregnation solution CaCl_2_/glycerol/ethanol to obtain CC/SA/TP compounds with antibacterial, antioxidant, and hemostatic functions, and the porous structure of CC/SA/TP can regulate wound bleeding and may be applied to wound dressings.

Wen et al. [117] used electrospinning technology to prepare a new type of composite fiber using polyurethane (PU)/polyacrylonitrile (PAN)/tea polyphenols (TPs) as raw materials, and found that the 120 μm composite fiber could continuously release TPs, which had good antibacterial properties against Gram-positive and Gram-negative bacteria, and scavenged DPPH free radicals. Based on its excellent performance, it is used in wound dressings and tissue catheters. Wang Chaoyang et al. [118] made a polyacrylic acid/polyacrylamide/tea polyphenol/Yunnan Baiyao gelatinous powder, and the hydrogel had good antibacterial properties against Staphylococcus aureus and *Escherichia coli* in the antibacterial experiment. In in vitro coagulation, a large number of red blood cells accumulate on the surface of the hydrogel to accelerate blood clotting. Based on the anti-inflammatory properties and cell proliferation of tea polyphenols and Yunnan Baiyao, biomaterials can be used to treat acute bleeding and promote wound healing. Jinhua et al. [119] used polylactic acid–glycolic acid (PLGA) nanoparticles to load tea polyphenols, and then coated platelet membranes (PMs) on the surface of nanoparticles, and the nanoparticles were targeted to accumulate in lung and vascular endothelial cells in vivo, which can reduce the secretion of inflammatory cytokines, and this biomimetic nanomaterial is expected to be applied to treat infectious diseases.

### 3.5. Heparin

Heparin is a mucopolysaccharide sulfate, commonly used as an anticoagulant in clinical practice, which can inhibit the binding of platelet surface receptors to thrombin substances, reduce the aggregation and adhesion of platelets, and reduce the risk of thrombosis. Heparin can bind to antithrombin III, enhance the affinity and inhibition of antithrombin III and coagulation factors, and accelerate the inactivation process of coagulation factors. The grafting of heparin to the surface of hollow fibrous membranes modifies the adsorption of surface proteins, resulting in reduced platelet adsorption [120]. Heparin significantly promotes the inactivation of antithrombin III on thrombin and inactivates FXa, FIIa, and FVIIa to inhibit blood clotting. In addition, it stimulates the release of fibrinolytic and anticoagulant substances from the vascular endothelium by activating protein C, and regulates tissue plasminogen activators to solubilize the fibrin network. Heparin has been reported to bind to pro-inflammatory molecules on the cell surface, such as P-selectin, intercellular adhesion molecule-1 (ICAM-1), and integrin macrophage-1 antigen (Mac-1) on the cell surface, through electrostatic interactions, thereby inhibiting activated coagulation-assisted inflammation and preventing receptor-mediated pro-inflammation to reduce the risk of thrombosis [121].

Yu Wen et al. [122] deposited chitosan and heparin on the surface of 316L stainless steel through layers of self-assembly technology to form a chitosan/heparin composite coating, which prolonged thrombin time and thromboplastin time to reduce the formation of thrombosis. Liu Xingyu et al. [123] found that a heparin/chitosan coating can reduce the adhesion of Staphylococcus aureus and inhibit the formation of stones in ureteral stents by modifying the ureteral surface and retrograde. Bukola O Awonusi et al. [124] applied heparin nanoparticles to the surface of Zn-Cu alloy for modification, which can be used for urinary system implantation, and reduce bacterial infection caused by implants and reduce the occurrence of inflammation.

**Figure 5:**
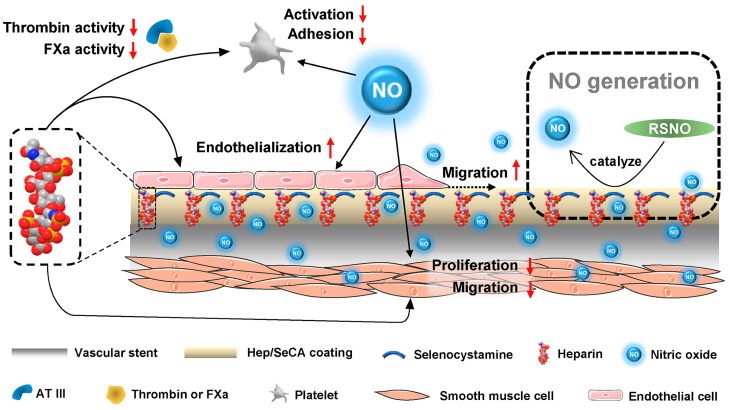
Biomimetic engineering of an endothelium-like coating through the synergic application of bioactive heparin and nitric oxide-generating species. The endothelium–biomimetic coating imparts the modified cardiovascular stent with the ability to combine the physiological capabilities of both heparin and NO, which creates a favorable microenvironment for inhibiting the key components in the coagulation cascade, such as Factor Xa and thrombin (Factor IIa) and platelets, as well as the growth of ECs over SMCs. These features endow the vascular stent with the abilities to impressively improve the antithrombogenicity, induce re-endothelialization, and prevent restenosis in vivo [129].

Polyetheretherketone (PEEK) can be used in orthopedic and dental alternatives to simulate the mechanical properties of bone, with good biocompatibility and good chemical stability [125]. Zhang Wenning et al. [126] prepared heparinized hydrogels by the free radical cross-linking polymerization (FRCP) of heparin and polydopamine. According to the catechol portion of the PDA, O_2_ redox is converted to H_2_O_2_, and heparin creates acidic conditions that favor the conversion of H_2_O_2_ to -OH, enhancing the combined sterilization of cations and ROS. Through antimicrobial experiments, it was found that the presence of these two improved the antimicrobial activity of hydrogels, which was applied to implants with antithrombotic and antibacterial properties. Jin Yingying et al. [127] conjugated anticoagulant heparin (Hep) and antibacterial carboxymethyl chitosan (CMCS) to the surface of polydopamine (PDA)-coated polyurethane (PU) membrane, namely PU/PDA-Hep/CMCS, and the amine and carboxyl groups in CMCS can bind to the bacterial surface, destroy the cell membrane, induce the leakage of components in the package, and lead to cell death. HEP can promote the binding of antithrombin and thrombin involved in activating factors to exert antibacterial effects. CMCS implantable materials can significantly reduce the activity of NF-kB and down-regulate inflammatory cytokines in rabbit models. In in vitro antimicrobial experiments, CMCS implants had good antibacterial properties against *Escherichia coli* and Staphylococcus aureus. Wang et al. [128] synthesized a molecular layer composed of sodium carboxymethyl cellulose sulfate (SCMC) and chitosan (CS) on the surface of polylactic acid through layer-by-layer self-assembly method, and the modified polylactic acid membrane has good cytocompatibility, antibacterial and anti-inflammatory abilities, and can be used for the surface modification of cardiovascular implantation. Hua et al. [129] designed a smart biomimetic coating which is modified with heparin and can release NO (Figure 5). Using heparin anticoagulation and NO release to inhibit the proliferation of smooth muscle cells, the biomimetic coating can improve the microenvironment of endothelial cells, and the application to vascular stents can inhibit the formation of thrombosis and resist stenosis.

### 3.6. Propolis

**Figure 6:**
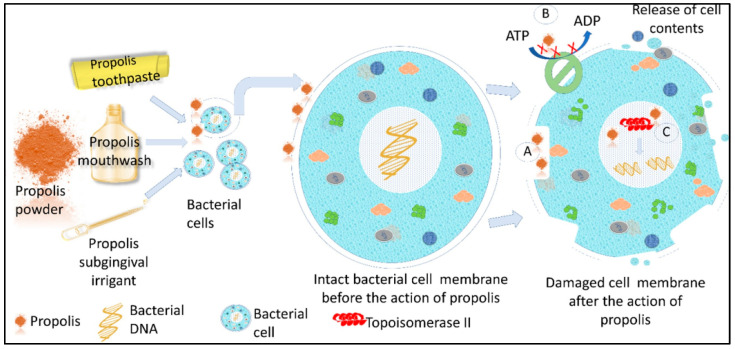
The mechanisms of antibacterial action of propolis—(A) propolis causes damage to the cell membrane, leading the cell contents to leak out, causing cell lysis. (B) Propolis inhibits adenosine triphosphate (ATP) formation, inhibiting mobility and the metabolism of the cell, impeding cell function (C) Propolis inhibits topoisomerase activity, causing DNA damage and mitotic failure [131].

Propolis is a natural ingredient consisting of a resinous mixture of honey compounds of various plant origins. Propolis contains a large amount of flavonoids, polyphenols, and other organic substances; propolis can change the permeability of cell membranes and lead to the leakage of cell contents, reduce energy metabolism, inhibit RNA polymerase to inhibit protein synthesis [130], and can also inhibit adenosine triphosphate (ATP) to hinder cell activities to exert antibacterial effects [131]. The antibacterial mechanism of propolis is shown in Figure 6.

Using the extract of propolis (EEP) as a raw material and mixing with silver nitrate to synthesize silver nanoparticles, sodium alginate was used as a fixative to fix the propolis–silver nanoparticles on the surgical suture line to prevent infection at the surgical site. According to the good antibacterial activity of propolis, propolis can be applied to the surface of porous polyurethane material to form a hydrogel, which can be used as a wound dressing to promote wound healing [132]. Propolis can effectively inhibit the proliferation of bacteria; the surface of wollastonite can produce hydroxyapatite, which can improve the viability of cells, and the use of propolis-modified wollastonite (CaSiO_3_) 3D-printed scaffolds can be used to repair bone damage [133]. Propolis was loaded onto TiO_2_ nanotubes (TNT), and it was found that the TNT loaded with propolis could reduce the expression of inflammatory factors IL-1β and TNF-α, and increase the expression of fibrinogen and osteogenic protein differentiation proteins BMP-2 and BMP-7 by staining in a rat mandible model [134].

### 3.7. Chitosan

Chitosan extract usually comes from the shell of crustaceans such as shrimp and crab, and chitosan is a natural polycationic compound formed by the deacetylation of chitin, which has good biocompatibility and unique film-forming properties under the action of an electric field, enabling osteoblasts to adhere and grow on the surface of the coating [135]. Chitosan is often used as a surface coating for implants, wound dressings, stents, etc., in biomaterial coatings to make better use of the biological activity of chitosan. A kind of chitosan was designed to prepare a silica–chitosan coating by the sol–gel method; the increase in chitosan content could improve the antibacterial property, and the release of silicon could promote the formation of bone [136]. The anode is made of platinum wire, the cathode is made of titanium or 316L stainless steel, the chitosan powder is dissolved in dilute hydrochloric acid, mixed with ethanol of different qualities to form an electrolyte, and a hydrophilic chitosan coating is formed by electrophoretic deposition, which has enhanced corrosion resistance with bare metal, and chitosan can be applied to the anti-corrosion of orthopedic implants [137]. Using the above cathode and anode, the electrolyte is replaced with chitosan and carbon nanotubes (MWCNTs) to prepare a hydrophilic MWCNT/chitosan coating, which can efficiently enrich calcium in the simulated body fluid, promote the formation of surface apatite layer, and can be applied to orthopedic implants to prevent corrosion [138]. Alkali heat treatment is beneficial to the combination of the negatively charged hydrate (HTiO_3_·H_2_O) on the surface of titanium and the cationic functional group (NH_3_^+^) of chitosan, and then the titanium is soaked in chitosan/ZnO solution for 6 h to form a composite coating; the formation of the composite coating inhibits the growth of *E. coli* and the adhesion of bacteria, and with the increase in zinc oxide content, the release of Zn^+^ can combine with the negatively charged bacteria and destroy the structure, the antibacterial property is enhanced, and the compatibility with MG-63 cells is good. It can be applied to orthopedic and dental implants [139]. Khoshnood N et al. [140] used fused deposition modeling 3D-printing technology to prepare polycaprolactone/chitosan composite coating on the surface of AZ21 magnesium alloy, the corrosion resistance of the composite coating was improved, and it had antibacterial activity against both Gram-negative and -positive bacteria; the abundant functional groups on the surface of chitosan could promote the binding of cells to the scaffold surface, and the presence of chitosan on the surface of ARS staining could lead to the deposition of calcium. RT-PCR was used to detect the content of osteogenic markers (COLI [141], ALP [142], RUNX2 [143]), and it was found that the composite scaffold increased the content of osteogenic markers and promoted bone regeneration at 7 days/14 days. Therefore, 3D-printed composite scaffolds can be used for the treatment of bone defects, regeneration and repair.

**Figure 7:**
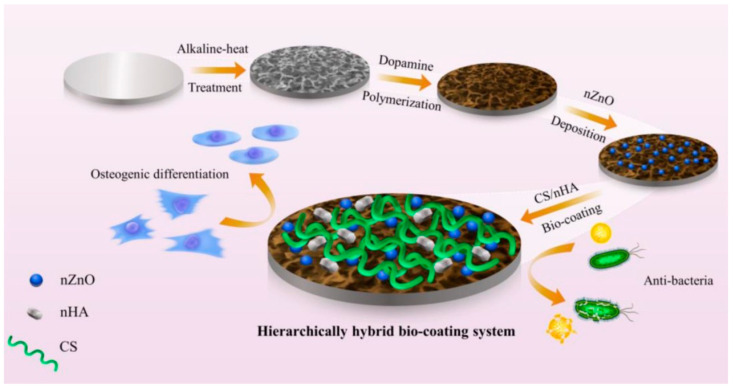
Hierarchically hybrid biocoatings on Ti implants are developed by gradual incorporation of polydopamine (PDA), ZnO nanoparticles (nZnO), and chitosan (CS)/nanocrystal hydroxyapatite (nHA) via oxidative self-polymerization, nanoparticle deposition, solvent casting and evaporation methods for enhancing their antibacterial activity and osteogenesis [146].

Zheng Weishi et al. [144] used layer-by-layer self-assembly technology to deposit carboxymethyl chitosan, gelatin, and alginate on the surface of cotton yarn in turn to form a new type of composite dressing, and the experimental results of a mouse-liver injury model and mouse-tail docking model showed that the composite dressing had a good hemostatic effect and could promote wound healing, which was better than that of medical cotton gauze. Gao Xiang et al. [145] used a Michael addition reaction to prepare polylactic acid nanofibers with maleilated chitosan/thiolated hyaluronan composite coatings. In a diabetic mouse model, it was shown that wound healing with the composite coating is faster, and can promote angiogenesis and collagen deposition. Wang et al. [146] sequentially deposited polydopamine (PDA), ZnO nanoparticles (nZnO), and chitosan (CS)/nanocrystalline hydroxyapatite (nHA) on a titanium substrate for a bioactive coating (as shown in Figure 7). Nanocrystalline hydroxyapatite (nHA) improves the surface structure and wettability of titanium implants and inhibits the growth of bacteria. Mixed chitosan can improve cytocompatibility and promote osteoblast differentiation. Based on the antimicrobial properties, and promoting cell osteogenic differentiation, composite coatings have great potential in orthopedics and dentistry.

### 3.8. Other Applications

There are many types of natural medicines, and there are countless applications in the coating of biomaterials. The main active factor of Salvia miltiorrhizae, Tanshinone IIA(TAN), has anti-inflammatory [147], antioxidant [148], and anti-apoptotic effects [149]. TAN delivery silk-fibroin scaffolds are created by freeze-drying combined with the silk-fibroin self-crosslinking method. The prepared TAN delivery silk-fibroin scaffold can promote the activity of chondrocytes, regenerate cartilage tissue, reduce oxidative stress, and can be used to repair cartilage defects [150]. Astragalus polysaccharides in Astragalus membranaceus have anti-inflammatory [151] and angiogenic effects [152], and a nanofiber membrane composed of gelatin and Astragalus polysaccharides was prepared by electrospinning technology [153], and the release of Astragalus polysaccharides can exert anti-inflammatory effects, promote the generation of blood vessels, and can be used as wound dressings to promote wound healing. Ginkgolide is extracted from ginkgo biloba, which has antioxidant [154], anti-inflammatory [155], and proliferative properties [156], and uses hyaluronic acid and ginkgolide to form a hydrogel, which can reduce inflammation and enhance the production of blood vessels, and can be used for diabetic wound healing and promoting tissue regeneration [157]. Madian Noha G et al. [158] used chitosan mixed with different concentrations of cellulose/honey/curcumin to improve its properties, and found that the CS-Cur cross-linked film had better mechanical properties and the best antibacterial effect, which could be used as a wound dressing. Electrospun composite fibers (PCL-COL-CUR) are prepared by incorporating curcumin into solutions of polycaprolactone (PCL) and collagen (COL), which can be used as antimicrobial dressings [159].

## 4. Challenges and Recommendations

With the rapid development of the biomedical field, biomaterial coatings are playing an increasingly important role in improving the performance of medical devices, promoting tissue repair, and preventing infection. Natural medicines have become a research hotspot in the field of biomaterial coatings due to their unique biological activity, low toxicity, and convenient accessibility, and common natural medicines such as berberine, allicin, chitosan, collagen, etc. can be loaded onto the surface of biomaterials by the physical adsorption method, chemical grafting method, layer-by-layer self-assembly method, the sol–gel method, and other methods to form a coating with specific functions. For example, loading the surface of orthopedic implants with natural drug coatings can promote osseointegration and reduce the risk of infection. Loading natural drugs with anticoagulant effects on cardiovascular and cerebrovascular stents can reduce the risk of thrombosis. Wound dressings are loaded with tissue-promoting drugs to accelerate wound healing and tissue regeneration.

However, the application of natural medicines to biomaterial coatings is not smooth sailing, and there are still many difficulties. The chemical structure of natural medicines is generally complex, and it is easy to be degraded or deteriorated by external factors such as light, humidity, temperature, etc., resulting in changes in biological activity. Processing conditions such as high temperature, pH, organic solvents, etc., during the preparation of coatings can cause damage to the structure and activity of natural medicines. When and how much natural medicine is released from the coating is also a major issue, as a release that is too fast may lead to side effects caused by high local concentrations, a release that is too slow may not achieve an effective effect, and bioenvironmental factors will also increase the difficulty of release. The strength of the bond between the coating and the substrate material is also critical, as if the bond is not strong, the coating will easily fall off, and if it is too strong, it will block the release of the drug load. Natural medicines have good biocompatibility, but questions remain, such as whether they will cause immune reactions or allergic reactions when they are prepared on the surface of the material and implanted in the body, whether they will disrupt certain balances in the body for a long time, and whether degradation products will cause a series of reactions in the body. The application of natural medicine involves the innovative application of medical devices, and the corresponding laws and regulations, preparation processes, and testing methods are not perfect, clinical trials may be few, and product industrialization has not yet been realized. A series of questions are raised from the negative aspects of the stability, biological activity, drug release, durability, and biocompatibility of natural medicines.

In view of the series of problems raised, future development should focus on solving these problems. With the advancement of science and technology and biotechnology, many new natural medicines have been found to have potential application value, expanding the source of new drugs. In addition, genetic engineering, synthetic biology and other methods can be used to optimize and transform natural medicines to improve biological activity and stability. The deep cross-integration of multiple disciplines promotes the new technological breakthrough of natural medicine in biomaterial coatings. For example, the application of nanotechnology can achieve precise loading and controlled release of natural medicines. The development of smart responsive materials enables coatings to automatically regulate drug release in response to changes in the in vivo environment; 3D-printing technology enables the preparation of coatings with complex structures and individualization. With the development of the concept of precision medicine, the future of biomaterial coatings will pay more attention to personalized customization. Depending on the patient’s individual differences, such as the type of disease, severity of the disease, immune status, etc., the appropriate natural medicine and coating formula are selected to achieve the best treatment effect. At the same time, bioinformatics and big data analysis are used to provide patients with more accurate treatment plans. The preparation of natural medicine coatings should closely follow current development practices, focus on environmental protection and sustainability, reduce the waste of resources in the preparation process, and make more use of drug carriers of renewable resources, as well as improving the corresponding laws and regulations, preparation methods, and testing processes, increasing the number and quality of clinical trials, and slowly realizing the commercialization of products. It is of great significance to strengthen basic research and deeply explore the mechanism of action of nature. Through the study of cells and molecules, the relationship between the biological activity of natural drugs and the performance of coatings is revealed, and a theoretical basis is provided for the development of more efficient and safe coatings.

The application potential of natural medicine in biomaterial coating is huge, but it also faces some challenges. Through technological innovation, cross-integration between disciplines, the improvement of corresponding laws and regulations, strengthening the in-depth basic research, and other measures, the problems faced will be slowly solved, in order to achieve the wide application of natural medicine coating in the field of biomedicine and to contribute to the cause of human health. In the future, there will be more innovative natural medicine coatings for patients, to provide better treatment and a higher quality of life.

## 5. Conclusions

Natural medicines are abundant, with a wide range of sources, low toxicity, and good biological activity. The application scale of biomaterials has been popularized in all aspects of life, such as the manufacturing of artificial joints, heart valves, vascular stents in medical devices, etc. Biomaterials are used as drug carriers to achieve controlled release and targeted delivery of drugs. They are also used to construct tissue-engineering scaffolds, etc. However, a series of problems, such as the service life and biocompatibility of materials in different physiological environments, will have a certain impact. Therefore, the combination of natural medicine and biomaterials forms a protective barrier to further improve the performance and biocompatibility of medical devices. For example, curcumin and allicin on the surface of medical devices can reduce the risk of bacterial infection and inflammation. Heparin is encapsulated into a hydrogel to achieve a controlled drug release.

This review starts with the definition of natural medicine and forms coatings by using different methods according to the properties of natural medicines. The biological activity of common drugs is detailed, and many natural medicines are widely studied and used in orthopedic implants, vascular stents, wound dressings, and drug delivery. Although natural medicines have made some progress in the application of biomaterial coatings, they still face many challenges, such as the binding strength between coatings, the controllability of drug release in drug-loading systems, and biocompatibility, and a series of questions such as clinical application and whether mass production can be achieved. Constructive suggestions are made on these issues. The application of natural medicines and biomaterials will continue to be expanded in the future, and more functional coatings will be innovated upon, which will be applied to all walks of life to improve the lives, health, and quality of life of human beings.

## Figures and Tables

**Table 1:** Chemical grafting.

Materials	Reaction Process	Reference
Glycidyl methacrylate (GMA), carbon fiber (CF)	The COOH on the surface of CF would lose electrons and then remove carbon dioxide to generate carbon radicals on the surface of CF. The carbon radical would attack the carbon–carbon double bond in GMA to initiate the radical polymerization of GMA monomers and graft polymers would be formed on the CF surface.	[45]
Waterborne polyurethane coatings (WPU), graphene oxide (GO)	The hydroxyl group on the surface of GO reacts with the amino group of the WPU molecular chain to form an amide bond.	[46]
Graphene oxide (GO), Ethylenediamine (EDA), polyvinylidene fluoride (PVDF)	GO-NH2 obtained by GO and EDA by a coupling reaction was grafted with dehydrofluorinated PVDF (DF-PVDF) in a Michael addition reaction.	[47]
Carboxymethyl cellulose (CMC), epigallocatechin gallate (EGCG-*g*-CS)	Polyionic complexes were formed by ionic bonding of the amino groups in EGCG-*g*-CS with the carboxyl groups in CMC.	[48]
Epsilon-poly-l-lysine (EPL), cellulose	Hydroxyl groups on C6 of cellulose were oxidized to carboxyl groups by TEMPO, and a grafting reaction was achieved between newly formed carboxyl groups of cellulose and amino of EPL.	[49]

## Data Availability

No new data were created or analyzed in this study.
